# Neo-adjuvant *ch*emotherapy followed by surgery *versus* surgery *a*lone in high-*ris*k patients with resectable colorectal liver *m*et*a*stases: the CHARISMA randomized multicenter clinical trial

**DOI:** 10.1186/s12885-015-1199-8

**Published:** 2015-03-26

**Authors:** Ninos Ayez, Eric P van der Stok, Hans de Wilt, Sandra A Radema, Richard van Hillegersberg, Rudi M Roumen, Gerard Vreugdenhil, Pieter J Tanis, Cornelis J Punt, Cornelis H Dejong, Rob L Jansen, Henk M Verheul, Koert P de Jong, Geke A Hospers, Joost M Klaase, Marie-Cecile Legdeur, Esther van Meerten, Ferry A Eskens, Nelly van der Meer, Bruno van der Holt, Cornelis Verhoef, Dirk J Grünhagen

**Affiliations:** 1 Department of Surgical Oncology, Erasmus MC Cancer Institute, Groene Hilledijk 301, 3075 EA Rotterdam, The Netherlands; 2 Department of Surgical Oncology, Radboud University, Nijmegen Medical Center, Nijmegen, The Netherlands; 3 Department of Medical Oncology, Radboud University, Nijmegen Medical Center, Nijmegen, The Netherlands; 4 Department of Surgery, University Medical Center Utrecht, Utrecht, The Netherlands; 5 Department of Surgery, Máxima Medical Center, Veldhoven, The Netherlands; 6 Department of Medical Oncology, Máxima Medical Center, Veldhoven, The Netherlands; 7 Department of Surgery, Academic Medical Center, Amsterdam, The Netherlands; 8 Department of Medical Oncology, Academic Medical Center, Amsterdam, The Netherlands; 9 Department of Surgery, Maastricht University Medical Center, Maastricht, The Netherlands; 10 Department of Medical Oncology, Maastricht University Medical Center, Maastricht, The Netherlands; 11 Department of Medical Oncology, VU University Medical Center, Amsterdam, The Netherlands; 12 Division of Hepato-Pancreato-Biliary Surgery and Liver Transplantation, Department of Surgery, University Medical Center Groningen, University of Groningen, Groningen, The Netherlands; 13 Department of Medical Oncology, University Medical Center Groningen, Groningen, The Netherlands; 14 Department of Surgery, Medisch Spectrum Twente, Enschede, The Netherlands; 15 Department of Internal Medicine, Medisch Spectrum Twente, Enschede, The Netherlands; 16 Department of Medical Oncology, Erasmus MC Cancer Institute, Rotterdam, The Netherlands; 17 Clinical Trial Center, Erasmus MC Cancer Institute, Rotterdam, The Netherlands

**Keywords:** Colorectal liver metastases, Neo-adjuvant chemotherapy, Surgical resection, Clinical risk score

## Abstract

**Background:**

Efforts to improve the outcome of liver surgery by combining curative resection with chemotherapy have failed to demonstrate definite overall survival benefit. This may partly be due to the fact that these studies often involve strict inclusion criteria. Consequently, patients with a high risk profile as characterized by Fong’s Clinical Risk Score (CRS) are often underrepresented in these studies. Conceptually, this group of patients might benefit the most from chemotherapy. The present study evaluates the impact of neo-adjuvant chemotherapy in high-risk patients with primary resectable colorectal liver metastases, without extrahepatic disease. Our hypothesis is that adding neo-adjuvant chemotherapy to surgery will provide an improvement in overall survival (OS) in patients with a high-risk profile.

**Methods/Design:**

CHARISMA is a multicenter, randomized, phase III clinical trial. Patients will be randomized to either surgery alone (standard treatment, arm A) or to 6 cycles of neo-adjuvant oxaliplatin-based chemotherapy, followed by surgery (arm B). Patients must be ≥ 18 years of age with liver metastases of histologically confirmed primary colorectal carcinoma. Patients with extrahepatic metastases are excluded. Liver metastases must be deemed primarily resectable. Only patients with a CRS of 3–5 are eligible. The primary study endpoint is OS. Secondary endpoints are progression free survival (PFS), quality of life, morbidity of resection, treatment response on neo-adjuvant chemotherapy, and whether CEA levels can predict treatment response.

**Discussion:**

CHARISMA is a multicenter, randomized, phase III clinical trial that will provide an answer to the question if adding neo-adjuvant chemotherapy to surgery will improve OS in a well-defined high-risk patient group with colorectal liver metastases.

**Trial registration:**

The CHARISMA is registered at European Union Clinical Trials Register (EudraCT), number: 2013-004952-39, and in the “Netherlands national Trial Register (NTR), number: 4893.

## Background

### Colorectal liver metastases: surgical treatment

Colorectal cancer (CRC) is one of the leading causes of cancer death. It is in the top 3 most commonly diagnosed cancers, with over 1.2 million new cases and over 600,000 deaths estimated to have occurred in 2008 worldwide [1]. In approximately 20% of patients distant metastases are present at time of diagnosis [2]. The liver is the most common metastatic site. Approximately 50% of patients with early-stage disease will eventually develop colorectal liver metastases (CRLM) [3,4].

When metastases of CRC patients are restricted to the liver, possible curative treatment can be obtained by surgical resection. Complete surgical resection of CRLM improves 5-year survival rates to around 35-60% in selected patients [5-8]. However in only 10-20% of patients surgical resection of CRLM is feasible. Although surgery for CRLM provides the only potential for cure, cancer relapse is a common phenomenon, with a recurrence rate of up to 50% in the first 2 years after surgery [9].

### Chemotherapy for colorectal liver metastases

Initially, systemic treatment with 5-fluoruracil based regimens was standard of care in CRLM, improving OS from 6 to 10–12 months. The development of chemotherapeutic agents such as oxaliplatin and irinotecan has subsequently improved OS to a median of up to 24 months. Sequential treatment with all available cytotoxic agents, as well as the introduction of Epidermal Growth Factor receptor (EGFR) and Vascular Endothelial Growth Factor (VEGF) binding monoclonal antibodies have further increased overall survival [10-13].

The high relapse rate after curative resection of CRLM, and the efficacy of modern systemic treatment in the metastatic setting, have prompted investigators to perform numerous studies to evaluate the potential role of systemic chemotherapy combined with liver resection. The purpose of both adjuvant and neo-adjuvant chemotherapy is to treat microscopic disease that is not addressed by surgery. This microscopic disease may be promoting the high relapse rate that is observed after liver surgery [9]. Notably, current literature suggests that timing of additional chemotherapy (adjuvant vs. neo-adjuvant) seems to have no influence on outcome [14]. The role of perioperative chemotherapy in case of resectable CRLM was established in a randomized controlled trial [15]. In the mature OS analysis of this trial there was no significant effect on OS after a median follow up of 7 years [16].

### Stratification by clinical risk score

In the past, several clinical risk scores for the outcome of patients with CRLM have been published [7,17-25]. In 1999, Fong et al. described the most widely used CRS [19]. This prognostic scoring system has been verified by independent investigators [26]. Several authors have proposed the concept of stratification by CRS in relation to the effects of a multimodal treatment strategy on OS. These authors suggest that patients with a high risk score have a worse prognosis and might therefore benefit more from chemotherapy compared to patients with a low risk score [27-29].

These findings have prompted others and ourselves to retrospectively evaluate data on patients who have undergone liver resection for CRLM in the last decade with and without chemotherapy, stratified by CRS according to the Fong-criteria [30,31].

As described earlier, efforts to improve outcome of liver surgery by combining the resection with chemotherapy have failed to demonstrate definite OS benefit. This may partly be due to the fact that these studies often involve strict study protocol inclusion criteria. Consequently, patients with a high clinical risk score - which might benefit the most from chemotherapy - are often underrepresented in these studies. Since genuine survival benefit has not yet been demonstrated, could this low impact of chemotherapy on survival then be explained by the *relatively* low risk profile of the patients included in these trials?

### Study aim and hypothesis

The CHARISMA randomized clinical trial will evaluate the effect on OS of neo-adjuvant chemotherapy in patients with primary resectable CRLM and a CRS (Fong) of 3–5, thereby bearing a poor prognosis. The primary aim of this study is to compare OS in patients with resectable liver metastases randomized for treatment with chemotherapy, consisting of capecitabine and oxaliplatin (XELOX), followed by surgery versus surgery alone.

We hypothesize that neo-adjuvant chemotherapy will provide an improvement in OS in this high-risk patient group. Secondary endpoints in this study will be progression free survival (PFS), quality of life as assessed by QLQ-30 and MFI questionnaires, response to chemotherapy, morbidity of surgery and resection rate, and whether carcinoembryonic antigen (CEA) can predict for treatment response, PFS, and OS.

## Methods/Design

Patients with CRLM and a high CRS will be evaluated for inclusion by the local multidisciplinary team meeting. In this meeting, at least two surgeons with expertise in liver surgery should be present. In case of doubt, the imaging can be sent to a central expert panel. Patients are eligible for randomization if, in the opinion of a local expert panel, radical resection of the CRLM (R0-resection) is feasible.

**Figure 1:**
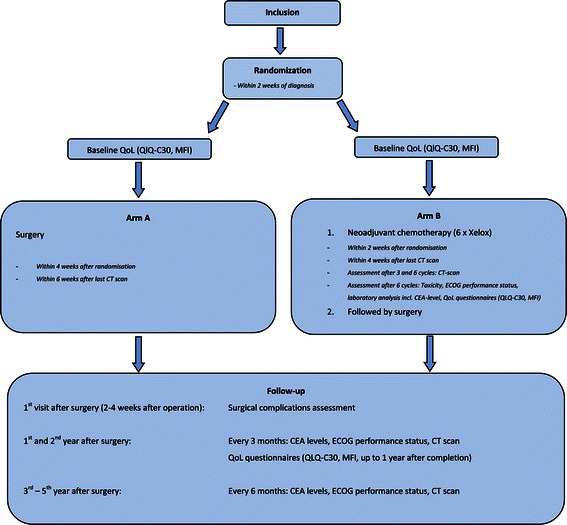
**Study flowchart.**

Patients will be randomized 1:1 to either (Figure 1):

- **Arm A:Surgery of the liver metastases**
- **Arm B:Neo-adjuvant oxaliplatin-based chemotherapy followed by surgery of the liver metastases**

### Study population

#### Inclusion criteria

Age ≥ 18 years, ECOG performance status 0–1. Histologically confirmed primary colorectal carcinoma. Radiological confirmed and primary resectable CRLM. CRS of 3–5 (Fong). Adequate bone marrow, liver and renal functions.

Before any study related procedure will be pursued, written informed consent must be given according to ICH/GCP and national/local regulations.

#### Exclusion criteria

Adjuvant chemotherapy for colorectal carcinoma given < 6 months prior to detection of the liver metastases. Prior non colorectal malignancies, except for basal or squamous cell carcinoma of the skin, or patients with carcinoma in situ of the cervix. Extrahepatic colorectal metastases. Locally advanced rectal cancer in situ requiring long-course pre-operative chemoradiotherapy. Major surgical procedures < 4 weeks prior to randomization. Pregnancy. History of psychiatric disability. Clinically significant cardiovascular disease. Uncontrolled hypertension. Lack of physical integrity of the upper gastro-intestinal tract, malabsorption syndrome, or inability to take oral medication. Known peripheral neuropathy. Organ allografts requiring immunosuppressive therapy. Serious, non-healing wound, ulcer, or bone fracture. Current or recent use of full-dose oral anticoagulants or thrombolytic agents for therapeutic purposes. Chronic treatment with corticosteroids. Serious intercurrent infections. Current or recent treatment with another investigational drug or participation in another investigational study. Psychological, familial, sociological or geographical conditions hampering compliance to the study protocol and follow-up schedule.

### Assessment of operability

All patients have to be screened by their treating surgeon for fitness to undergo liver surgery. In case of doubt, formal anesthetic assessment is mandatory prior to randomization.

### Assessment of resectability

Prior to resection of the CRLM, an expert panel must review imaging of patients enrolled in this study in order to determine resectability. Resectability is defined as the possibility to achieve R0 resection. The liver remnant should comprise a portal vein, a hepatic artery, and a bile duct, one of the three main hepatic veins. The liver remnant should have sufficient liver function and 2 segments free of metastases at the time of resection.

If these prerequisites cannot be met, radiofrequency ablation (RFA) is allowed to obtain resectability. However, RFA may only be used in combination with liver resection if the number of lesions to be treated with RFA does not exceed 3 and the largest diameter of these lesions is less than 3 cm.

### Therapeutic regimen of patients Arm A

Patients should preferably be randomized within 2 weeks of the definitive diagnosis of CRLM. Patients allocated to Arm A should preferably have their surgery within 4 weeks after randomization and within 6 weeks after the last CT scan. Adjuvant chemotherapy after R0 resection is not allowed. Protocol therapy ends following the liver resection.

### Therapeutic regimen of patients Arm B

Patients in Arm B will receive 6 cycles of XELOX. Oxaliplatin will be administered in a 130 mg/m^2^ dose, Capecitabine in a 1000 mg/m^2^ dose. Patients should preferably be randomized within 2 weeks of the definitive diagnosis of CRLM. Patients allocated to Arm B should start neo-adjuvant chemotherapy preferably within 2 weeks after randomization and within 4 weeks after the last CT scan. Treatment evaluation will occur after the 3^rd^ and 6^th^ chemotherapy cycle. In the case of progressive disease (PD) after the 3^rd^ cycle, a resectability check will take place. If patients remained resectable, they will be planned for surgery within 4–6 weeks after completion of the 4^th^ cycle. If patients are assessed to be irresectable, they will go off study protocol, but will be analyzed according to intention to treat principle.

After the last day of chemotherapy exposure, resection should take place at least 4 weeks, but at maximum 6 weeks later. Treatment evaluation can take place according to local hospital procedures, but should at least consist of a CT scan of the thorax/abdomen and CEA level. Adjuvant chemotherapy after R0 resection is not allowed. Protocol therapy ends following the liver resection.

### Endpoint

#### Primary endpoint

Primary endpoint of the study will be OS, calculated from the date of randomization to the date of death of the patient, from any cause. Patients still alive at the date of last contact will be censored.

#### Secondary endpoints

PFS will be defined from the date of randomization to the first event defined as local/distant recurrence or progression or death from any cause.

### Criteria of evaluation

Progressive or recurrent disease can be detected by imaging modalities (e.g. CT scan). A rise in serum tumor marker (e.g. CEA) is insufficient. In case of doubt, histological biopsy can provide definitive proof of progression/recurrence. Response to neo-adjuvant chemotherapy will be evaluated by CT scan using RECIST 1.1 criteria [32]. To evaluate the well-being of patients the European Organization for Research and Treatment of Cancer Quality of Life questionnaire (EORTC QoL) will be used. The EORTC QLQ-C30 is generally used to assess QoL of cancer patients; additionally the Multifactorial Fatigue Index (MFI) will be used. Toxicity will be graded according to the Common Terminology Criteria for Adverse Events (CTCAE) version 4.0. Surgical complications will be defined according to the standard classification of surgical complications [33]. Postoperative mortality will be defined as any death during hospitalization or within 30 days from surgery. Complication and post-operative mortality rates will be securely monitored and documented.

### Statistical considerations

#### Sample size and accrual

On the basis of retrospective data, we expect the hazard ratio (HR) for arm B to be 0.60. For the detection of a HR of 0.60 for the chemotherapy arm and with an expected 5-year OS of 25% in arm A, with two-sided significance level α = 0.05 and power 1 - β = 0.8, 126 deaths have to be reported before the final analysis will take place. This number of events is expected to be reached after the recruitment of 224 patients with an average accrual rate of 56 patients per year, and an additional follow up of 2 years. A HR = 0.60 corresponds to an increase of 5-year OS of 43% in arm B.

### Randomization

Eligible patients should be registered after written informed consent and before start of treatment (based on inclusion/exclusion criteria). Patients will be randomized for surgery versus neo-adjuvant chemotherapy followed by surgery in a 1:1 design. During randomization patients will be stratified by center, CRS score and status of primary tumor (still in situ vs. resected) with a minimization procedure, ensuring balance within each stratum and overall balance.

### Statistical analysis plan

The main analysis addressing the primary endpoint is planned after 126 events. No interim analysis is planned.

### Ethics

The study has ethical approval from the Erasmus MC medical-ethical committee. The study will be conducted in accordance with the ethical principles of the Declaration of Helsinki, the ICH-GCP Guidelines, the EU Clinical Trial Directive (2001/20/EG), and applicable regulatory requirements. The local investigator is responsible for the proper conduct of the study at the study site.

## Discussion

Currently, multimodal treatment is not incorporated in the standard of care for primary resectable colorectal liver metastases. To date, no definite evidence exists favoring administration of (neo) adjuvant chemotherapy in CRLM in addition to surgery. Considering the retrospective observations that pre-selection of patients by clinical prognostic characteristics may define a patient population expected to benefit from chemotherapy, CRS stratification provides the base for this randomized controlled trial.

Preceding studies of peri-operative chemotherapy combined with liver surgery often engaged strict study protocol inclusion criteria. Consequently, patients with a high CRS - which might benefit the most from chemotherapy - are often underrepresented in these studies. Possibly, this low impact of chemotherapy on survival could be explained by the *relatively* low risk profile of the patients included in these trials. Recently, two reports on patients with relatively low risk for recurrence have been published. Adam et al. performed an analysis of the LiverMetSurvey database on patients with solitary, metachronous, primarily resectable metastases. These patients have particularly favorable tumor biology and a low CRS. The authors concluded that these patients do not benefit from preoperative chemotherapy [34]. A recent systematic review of the literature by Lehmann et al. concludes that routine use of neo-adjuvant chemotherapy for patients with clearly resectable lesions limited to the liver is not recommended due to a lack of benefit on survival [35].

As mentioned before, several authors have proposed the concept of stratification by CRS with regard to the effects of systemic therapy. Tomlinson et al. demonstrated on actual 10-year survivors of liver surgery for CRLM that patients with a low CRS had a cure rate of 21% and that patients with a high CRS had a cure rate of 10% [27]. They suggest that this finding may be used to identify patients who might benefit from neo-adjuvant chemotherapy [27]. In a large, non-randomized study by Parks et al., adjuvant therapy did seem to improve OS [28]. In this study, patients with a high CRS had more benefit from adjuvant therapy than patients with a low CRS, again suggesting a role for CRS when considering chemotherapy.

These reports have stimulated others and our own unit to retrospectively evaluate data on patients that underwent liver resection for CRLM in the last decade with and without chemotherapy, stratified by CRS according to the Fong-criteria [19]. Rahbari et al. have evaluated the role of adjuvant chemotherapy in a cohort of 316 patients, of whom 43% were high-risk according to the “Memorial Sloan-Kettering Cancer Center CRS” (CRS > 2). They found that adjuvant chemotherapy had a profound impact on OS in the high-risk population (HR = 0.40), whereas in low-risk patients HR = 0.90 [31]. In a recent manuscript by Hirokawa et al. similar results are described with de use of adjuvant chemotherapy [36]. In our population of patients that underwent resection for CRLM in Rotterdam (N = 365), we have focused on neo-adjuvant chemotherapy. In this study, a pronounced improvement in OS was found in high-risk patients receiving neo-adjuvant chemotherapy versus no chemotherapy (median 67 months vs. 33 months, HR = 0.55 [95% CI 0.35-0.84], p = 0.006). This difference was absent in the low-risk group (median 65 months vs. 56 months, HR = 0.89 [95% CI 0.57-1.40], p = 0.62) [30]. Notably, these studies were retrospective and non-randomized. The sample size calculation of the present study is based on these retrospective data.

In a recent editorial by Jarnagin et al. it is suggested that future trials should strongly consider stratification by some scoring system [29], given the results of the retrospective studies as mentioned above. Our study will evaluate patients with resectable CRLM without extra hepatic disease and a CRS of 3–5 thereby bearing a poor prognosis. The primary aim of this study is to compare OS rates of patients with resectable liver metastases randomized for treatment with chemotherapy consisting of capecitabine and oxaliplatin (XELOX) followed by surgery, versus surgery alone. We hypothesize that adding neo-adjuvant chemotherapy to surgical resection of CRLM will provide an improvement in OS in patients with a high-risk profile. As secondary objectives we will study PFS, quality of life, treatment response on neoadjuvant chemotherapy, morbidity of surgery and resection rate, and whether CEA can predict for treatment response, PFS, and OS.

## Abbreviations

CEA: Carcinoembryonic antigen
CRC: Colorectal cancer
CRLM: Colorectal liver metastases
CRS: Clinical risk score
ECOG: Eastern cooperative oncology group
OS: Overall survival
PFS: Progression free survival
RCT: Randomized controlled trial
RFA: Radiofrequency ablation
XELOX: Chemotherapy consisting of capecitabine and oxaliplatin
